# Dissecting the functional differences and clinical features of R-spondin family members in metastatic prostate cancer

**DOI:** 10.18632/oncotarget.28758

**Published:** 2025-07-25

**Authors:** Aiden Deacon, Ava Gustafson, Allison Makovec, Ella Boytim, Gabriella von Dohlen, David Moline, Elin Kairies, Sam Kellen, Khalid Ishani, Megan L. Ludwig, Emily John, Alexis Anike, Hai Dang Nguyen, Scott M. Dehm, Justin M. Drake, Emmanuel S. Antonarakis, Justin Hwang

**Affiliations:** ^1^Department of Medicine, University of Minnesota-Twin Cities, Minneapolis, MN 55455, USA; ^2^Cornell College, Mount Vernon, IA 52314, USA; ^3^Department of Pharmacology, University of Minnesota- Twin Cities, Minneapolis, MN 55455, USA; ^4^Masonic Cancer Center, University of Minnesota-Twin Cities, Minneapolis, MN 55455, USA

**Keywords:** RSPO2, prostate cancer, Wnt signaling, genomics, therapeutics

## Abstract

This study investigates the R-spondin family of genes (*RSPO1/2/3/4*), a group of secreted proteins that act as Wnt regulators, and their subsequent role in advanced prostate cancer (PC). When evaluating transcriptomic data from primary and metastatic PC patients, we found that alterations in *RSPO2* were more prevalent than in other RSPO family members or Wnt-regulating genes *APC* and *CTNNB1*. Further, we found that *RSPO2* alterations in PCs were significantly associated with worse disease-free survival. Through our *in silico* modeling, RSPO2 exhibited strong positive associations with genes regulating epithelial-mesenchymal transition (EMT) and double-negative prostate cancer (DNPC), but had negative correlations with androgen receptor (AR) and AR-associated genes. Furthermore, 3D modeling of RSPO2 revealed structural differences between itself and other RSPOs. In cell lines, *RSPO2* overexpression caused up-regulation of EMT pathways, including EMT-regulatory transcription factors *ZEB1, ZEB2,* and *TWIST1*. Conversely, this was not observed when *CTNNB1* was overexpressed in the same models. These findings highlight that, in PC, RSPO2 functions as a unique member of the R-spondin family by promoting genes and signaling pathways associated with aggressive PC, and *RSPO2* amplifications are associated with poor outcomes in PC patients.

## INTRODUCTION

Prostate cancer (PC) remains the most diagnosed cancer and the second most lethal cancer in U.S. men [1]. PC is noted for its high mortality rate following progression to metastatic disease. Androgen deprivation therapy (ADT) targets the androgen receptor (AR) and is the standard of care for almost all patients with metastatic PC (mPC). However, most metastatic PC tumors continue to progress to become metastatic castration resistant prostate cancer (mCRPC), a disease stage that still requires novel interventions.

Several mechanisms of resistance have been implicated in the development or progression of mCRPC. While AR is the major driver of mCRPC, growing evidence indicates that pathways independent of AR, such as the Wnt pathway, are also relevant [2]. Compared to localized PC, mCRPC exhibits higher rates of alterations in genes encoding regulators of Wnt signaling, including inactivation of the negative regulator *APC* and stabilizing mutations or amplifications of the canonical transcription factor, *CTNNB1* [3]. Altogether, 15–20% of mCRPC patients harbored genomic alterations in the Wnt signaling pathway [4]. In both metastatic hormone-sensitive and castration-resistant PC, the presence of Wnt pathway alterations is associated with aggressive disease features and reduced survival [5, 6]. *CTNNB1* encodes β-catenin, a key Wnt transcription factor that promotes downstream signaling through known oncogenes such as Cyclin D-1 and c-Myc [7]. While *CTNNB1* is a high-value therapeutic target, it remains difficult to drug due to its nuclear localization and flat binding pockets [8]. Therefore, alternative approaches to target the activated Wnt pathway in mCRPC are appealing treatment strategies.

The R-spondin 2 (*RSPO2*) glycoprotein is one of the four members of the R-spondin family of genes. Thought to stabilize Wnt signaling, R-spondin 2 has been implicated in cell proliferation, migration, and invasion in other hormone-driven cancers, including ovarian cancer [9]. However, neither RSPO2 nor the rest of the family of R-spondins has been functionally examined in PC models. Our prior work, using unbiased computational approaches that model gene behavior, nominated *RSPO2* as a gene that promotes therapeutic resistance in mCRPC through unbiased computational approaches that model gene behavior [10]. In a seminal study of 150 mCRPC patients, activating pathogenic *RSPO2* structural rearrangements were identified, which led to gross overexpression of *RSPO2* transcripts in the tumor [4]. Outside of these findings, the role of RSPO2 and how it regulates progression and signaling in the PC cell has not been elucidated.

Here, we explored the correlation between RSPO2 and RSPO family members in mCRPC patients and in tissue culture models. *RSPO2* alterations lead to worse clinical outcomes in mCRPC and were correlated with the expression of genes and pathways known to promote metastasis and aggressive PCs resistant to ADT. Altogether, our findings nominate RSPO2 as a promising therapeutic target for mCRPC patients.

## RESULTS

### PC patients harbor more RSPO2 alterations compared to RSPO1/3/4 and Wnt signaling genes

**Figure 1:**
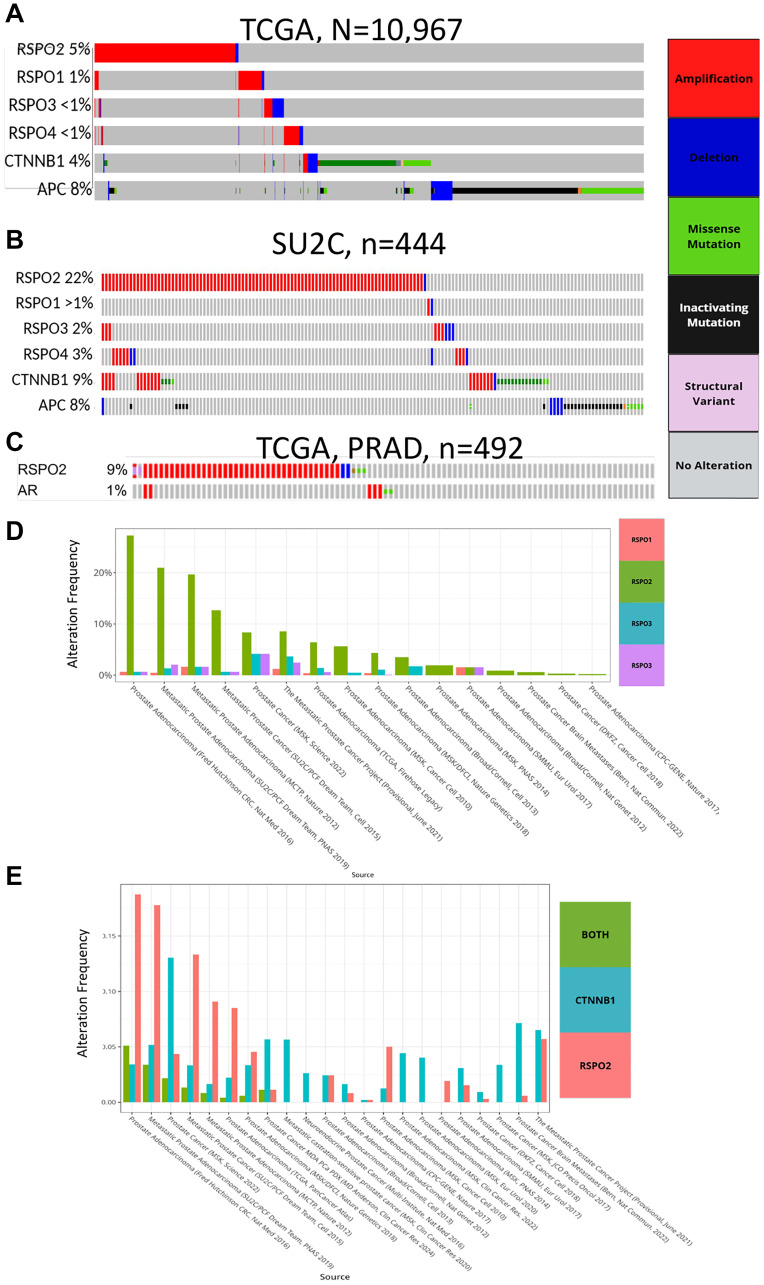
Through an oncoprint, genomic alterations in *RSPO1/2/3/4*, *CTNNB1*, and *APC* are depicted in (**A**) 10,967 tumor samples from the TCGA Pan Cancer Atlas, (**B**) 444 tumor samples from the SU2C study. (**C**) RSPO2 and AR alterations are shown based on the 492 samples in the TCGA prostate cancer study (PRAD). Across an aggregate of 16 PC datasets on cBioPortal (studies detailed in the x-axis), the prevalence of alterations is shown to compare (**D**) RSPO family members, (**E**) *RSPO2* amplifications, *CTNNB1* amplifications/mutations, or both.

To interrogate the clinical relevance of *RSPO2*, we examined the genomic alteration events and their prevalence in *RSPO2* and compared them to those in *RSPO1/3/4* and the Wnt-regulating genes *APC* and *CTNNB1*. Beginning with a pan-cancer analysis from The Cancer Genome Atlas (TCGA) (*N* = 10,967), we found that *RSPO2* alterations were present in 5% of cancer patients, with the majority being gene amplifications. Conversely, *RSPO1/3/4* were altered in less than 1% of patients. *CTNNB1* and *APC* alteration rates were comparable to *RSPO2* at 4% and 8%, respectively (Figure 1A). In 444 mCRPC patients from the Stand Up To Cancer 2019 (SU2C) study [11], we observed that 22% (*n* = 96) harbored *RSPO2* alterations, of which only 2 cases displayed *RSPO2* deletion. As in the pan-cancer analysis, *RSPO1/3/4* alterations remained at 3% or lower. About 9% of PC patients harbored *CTNNB1* alterations, whereas 8% had loss of *APC* (Figure 1B). In order to ascertain RSPO2’s relationship with AR, we investigated its role in primary prostate tumors (TCGA, *n* = 489) and observed *RSPO2* alterations in 9% of samples (Figure 1C). *RSPO2* was the most recurrently altered R-spondin family member in 15 out of 16 cohorts on cBioPortal [12] (Figure 1D). In order to discover the co-occurrence of the *CTNNB1* missense mutations, which are associated with the promotion of carcinogenic features [13], and RSPO2 alterations, we compared the frequency of *RSPO2* amplifications with *CTNNB1* missense mutations across 22 primary and metastatic PC patient cohorts from cBioPortal. As in the SU2C cohort, *RSPO2* amplification rates often exceeded the rate of *CTNNB1* alterations and co-occurred in 34% of cohorts (Figure 1E). Altogether, compared to *RPSO1/3/4* and other Wnt regulatory genes, *RSPO2* alterations were more prevalent in primary PC and mCRPC tissue specimens.

### RSPO2 amplifications are associated with worse clinical outcomes

**Figure 2:**
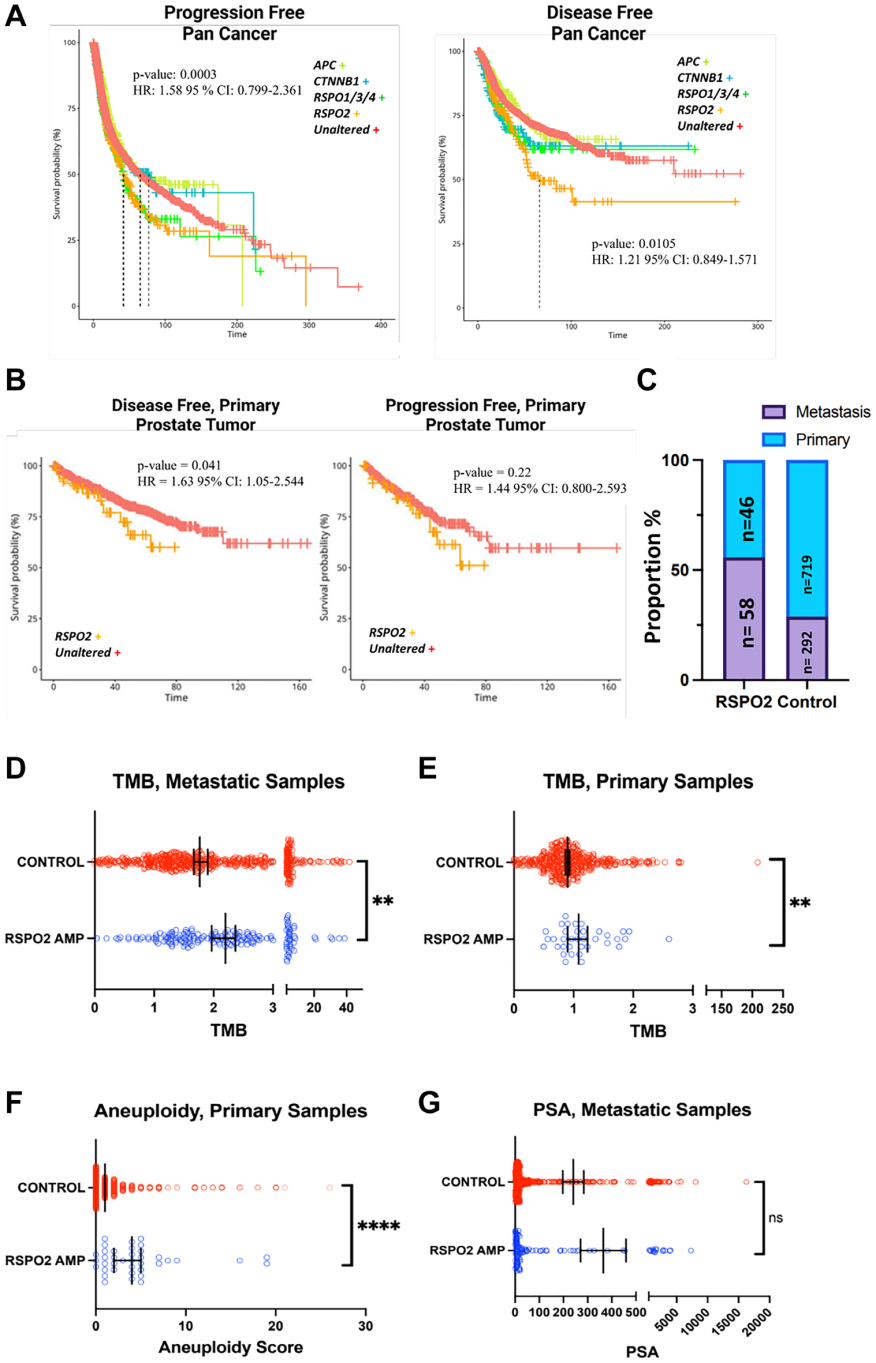
(**A**) Samples with annotated disease-free (*n* = 5,383) and progression-free (*n* = 10,613) survival are shown for cancer patients from the TCGA Pan Cancer Atlas based on if they have alterations in *RSPO2* (orange), *CTNNB1* (teal), *APC* (green), or the unaltered group (red). (**B**) TCGA PC samples were analyzed for disease or progression-free survival (*n* = 489) based on whether they had an *RSPO2* amplification (orange) as compared to the control (red) group. (**C**) In an aggregate of 16 PC studies, the proportion of tumors with and without *RSPO2* amplifications is depicted based on metastatic samples or primary samples. (**D**) TMB is shown for control or samples with *RSPO2* alterations based on mCRPC samples from cBioPortal (SU2C [33]). Of samples in the TCGA PC study, control and samples with *RSPO2* alterations are analyzed for their status in (**E**) TMB, (**F**) aneuploidy scores. (**G**) PSA levels are depicted for samples with or without *RSPO2* alterations based on an aggregate of metastatic prostate cancer samples (FHCRC [33, 35], SU2C, Eur Urol 2017 [34], PRAD Broad [36]).

Given the high prevalence of *RSPO2* amplifications in PC, we investigated the association of *RSPO2* amplifications with clinical outcomes. Across cancers, *RSPO2* amplifications exhibited the worst outcomes in both disease-free and progression-free survival (HR: 1.58 and 1.21, *p*-value: 0.0003 and 0.0105 CI: 0.799–2.361 and 0.849–1.571, respectively) (Figure 2A). In primary PC patients, as compared to unaltered groups, those with *RPSO2* amplifications trended towards worse disease-free survival, and exhibited significant differences in progression-free survival (HR = 1.44 and 1.63, *p*-value = 0.22 and 0.041, 95% CI: 0.800–2.593 and 1.05–2.544, respectively) (Figure 2B). Based on an aggregate of 16 PC datasets consisting of 1051 tumors, *RSPO2* amplifications (*n* = 104) were overrepresented in metastatic samples relative to primary tumor samples (54% vs. 28%, respectively) (Figure 2C). *RSPO2* amplifications were associated with significant increases in tumor mutational burden (TMB) in both primary (*p*-value = 0.0030) and metastatic (*p*-value = 0.0048) PC patients (Figure 2D, 2E). In primary PC patients, *RSPO2* amplifications were associated with increased aneuploidy score (*p*-value = <0.0001) (Figure 2F). *RSPO2* amplified patients exhibited trends of elevated serum prostate-specific antigen (PSA) levels (*p*-value = 0.66) (Figure 2G). Altogether, *RSPO2* amplifications exhibited worse outcomes across cancer and in PC. In PC, *RSPO2* amplified tumors harbored multiple clinical measurements of tumor malignancy.

### RSPO2 is functionally and structurally different than other R-spondins

**Figure 3:**
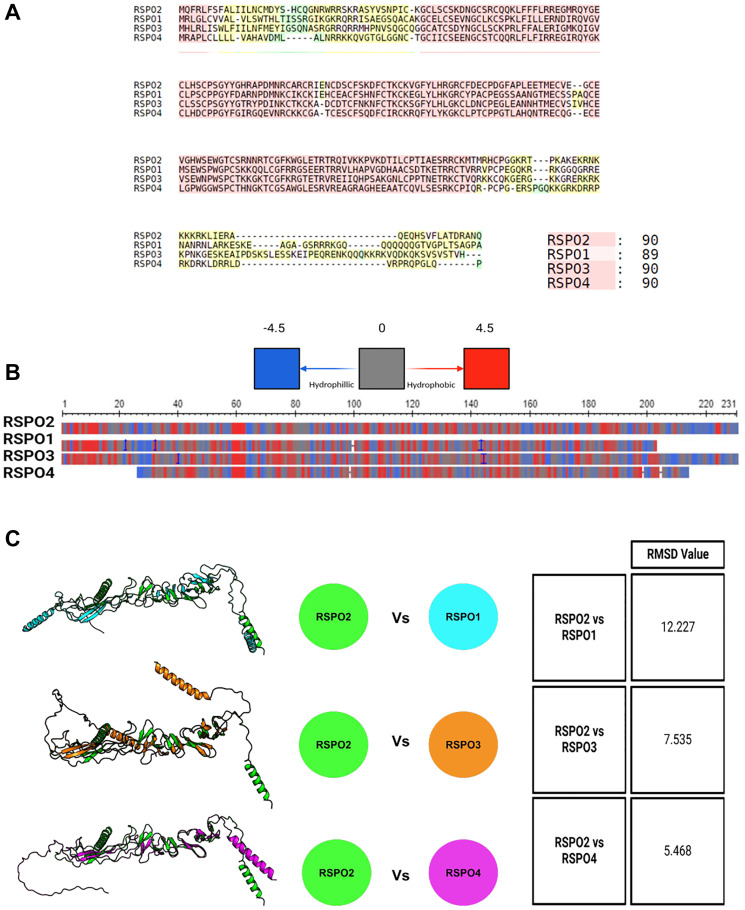
(**A**) The amino acid sequences are aligned, in which RSPO2 is compared to RSPO1/3/4. Similar (red), dissimilar (yellow), and unique regions (green) are shown. (**B**) General hydrophobicity of the amino acid side chains of RSPO2 is compared to RSPO1/3/4. The key indicates if a region is hydrophobic (red), hydrophilic (blue), or neutral (grey). (**C**) Alphafold2 was used to overlay the protein structure of RSPO2 with RSPO1/3/4. The similarity or differences were assessed based on RSMD scores.

Next, we compared RSPO family members based on amino acid sequence, hydrophobicity, and projected protein structure. Based on multiple sequence alignment, the amino acid sequences of R-spondin 2 and R-spondin1/3/4 had notable differences throughout the whole protein (Figure 3A). Based on relative hydrophobicity, we did not find clear differences based on these alignments (Figure 3B). Using Alpha Fold [14] and PyMOL [15], we compared the predicted protein structure similarity between R-spondin 2 and R-spondin 1/3/4. The root mean square deviation (RMSD), which measures the distance of Cα atoms between two superimposed residues in Angstroms (Å), was used to compare the predicted protein models. R-spondin 2, compared to R-spondin 1, showcased the most dissimilar predicted structure similarity (RMSD: 12.227), followed by R-spondin 3 (RMSD: 7.535), and R-spondin 4 (RMSD: 5.468) (Figure 3C). Overall, R-spondin 2 exhibited differences in both folding patterns and structure compared to the other three R-spondin proteins.

### RSPO2 activation is associated with genes encoding EMT regulators in mCRPCs

**Figure 4:**
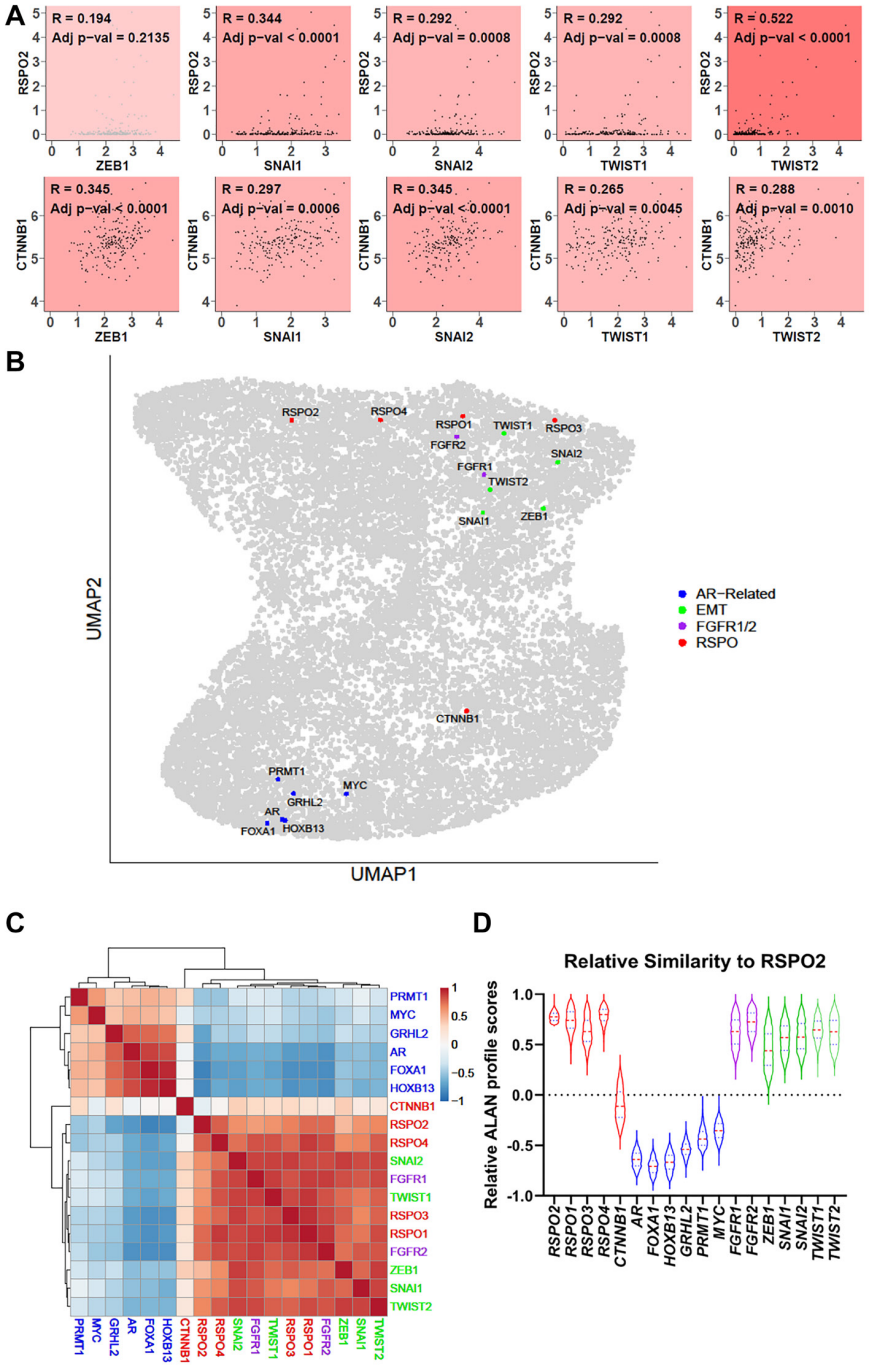
(**A**) In 208 SU2C mCRPC samples, Pearson correlations were used to compare the transcript expression of *RSPO2* or *CTNNB1* with EMT transcription factors, including *ZEB1*, *SNAI1*, *SNAI2*, *TWIST1*, and *TWIST2*. The coefficients and adjusted *p*-values are shown. (**B**) Outputs from an ALAN analysis are depicted in a UMAP in which each dot represents a gene. Here, genes related to AR (blue), EMT (blue), and FGFR (purple) are labeled along with RSPO2 (red). ALAN outputs for the same set of genes are depicted as **(C**) A hierarchical clustered heatmap (**D**) violin plots.

R-spondin 2 is a known regulator of EMT in gastrointestinal cancers [16]. Using the gene expression data from 208 mCRPC samples in the SU2C dataset, we examined the co-expression of *RSPO2* with regard to known epithelial-mesenchymal transition (EMT) transcription factors [17]. *RSPO2* exhibited overall positive correlation with *SNAI1* (R = 0.344, adj. *p* = <0.0001), *SNAI2* (R = 0.292, adj. *p* = 0.0008), *TWIST1* (R = 0.292, adj. *p* = 0.0008), with high *TWIST2* correlation (R = 0.522, *p* = <0.0001) (Figure 4A). We next used our published Algorithm for Linking Activity Networks (ALAN) [10] to compare the relative gene behavior of *RSPO2* with *AR* and other known *AR* cofactors, a panel of EMT genes, and genes that regulate androgen-independent growth, including *FGFR1/2* [18]. Based on the ALAN quantitative outputs, *RSPO2* behaved similarly to EMT-related genes and *FGFR1/2*, but exhibited opposing behavior with *AR* regulatory genes and *MYC*, a pan-cancer oncogene (Figure 4B–4D). Interestingly, while *CTNNB1* and *RSPO2* are both considered regulators of Wnt signaling, *RSPO2* exhibited far closer behavior to these EMT genes and *FGFR1/2*. These analyses indicated that *RSPO2* may be closely associated with EMT, a pathway that has consistently been associated with poor outcomes in patients with PC [19, 20].

### RSPO2 overexpression increases proliferation and alters the transcriptome in PC cell lines

**Figure 5:**
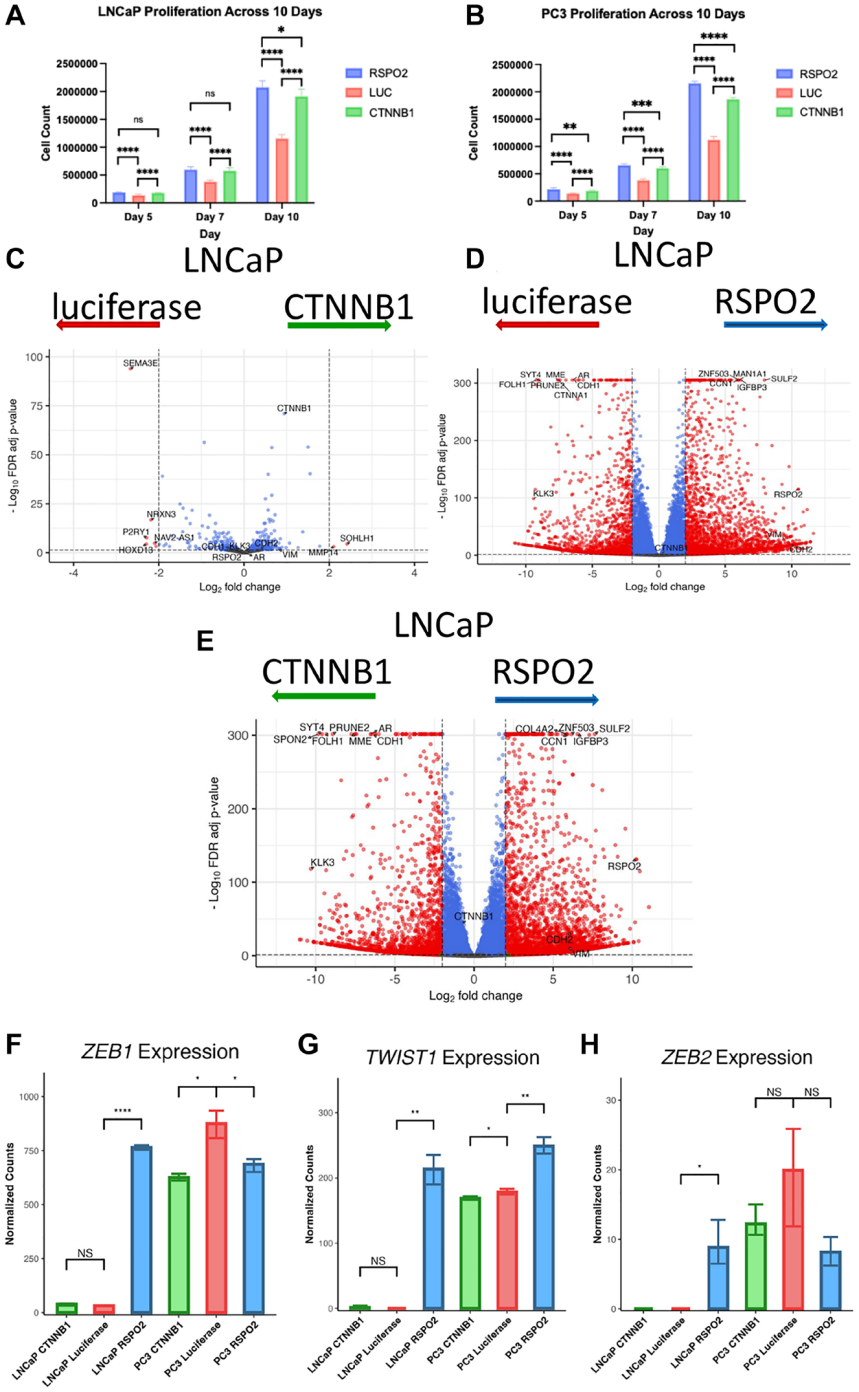
In 3 biological replicates each in triplicates, proliferation assays were performed over 10 days, in which the rates of (**A**) LNCaP (AR+) or (**B**) PC3 (AR-) cells that express either RSPO2, luciferase control (LUC), or CTNNB1 were shown at specific time points. ^****^ *p* < 0.0001. Differential gene expression profiles are shown in which we depict the differential expression of LNCaP cells with (**C**) CTNNB1 and LUC, (**D**) RSPO2 and LUC, (**E**) RSPO2 and CTNNB1. Of the same experiments, normalized counts are shown for relative expression of (**F**) ZEB1, (**G**) TWIST1, and (**H**) ZEB2. Normalized count statistical comparisons were conducted using Welch’s two sample *t*-tests, with ^*^ *p* < 0.05, ^**^ *p* < 0.01, ^***^ *p* < 0.001, ^****^ *p* < 0.0001.

To elucidate the functional impact of *RSPO2* in PC, we overexpressed *RSPO2*, *CTNNB1,* or a negative control (luciferase) in AR+ (LNCaP) and AR- (PC3) cell lines. In both PC cell lines, *RSPO2*-overexpressed cells exhibited increased proliferation relative to *CTNNB1*-overexpressed and negative control cells (Figure 5A, 5B). Additionally, based on RNA-sequencing done in biological triplicates, *RSPO2* overexpression led to profound alterations in the transcriptome when compared to luciferase or *CTNNB1* overexpression (Figure 5C–5E). Specifically, *CTNNB1* overexpression did lead to transcriptional changes, but the effects of *RSPO2* overexpression were substantially more robust at the transcriptome level when examining genes that were significantly up- or down-regulated. When examining the expression of the transcription regulators of EMT (namely *ZEB1, ZEB2,* and *TWIST1)*, *RSPO2* overexpression led to pronounced increases in *ZEB1* and *TWIST1*, an effect that was not found upon *CTNNB1* overexpression (Figure 5F–5H). Overall, *RSPO2* overexpression increased cell proliferation and uniquely enriched transcription factor expression involved in EMT regulation to a greater extent than *CTNNB1* overexpression.

### RSPO2 overexpression leads to upregulation of oncogenic signatures

**Figure 6:**
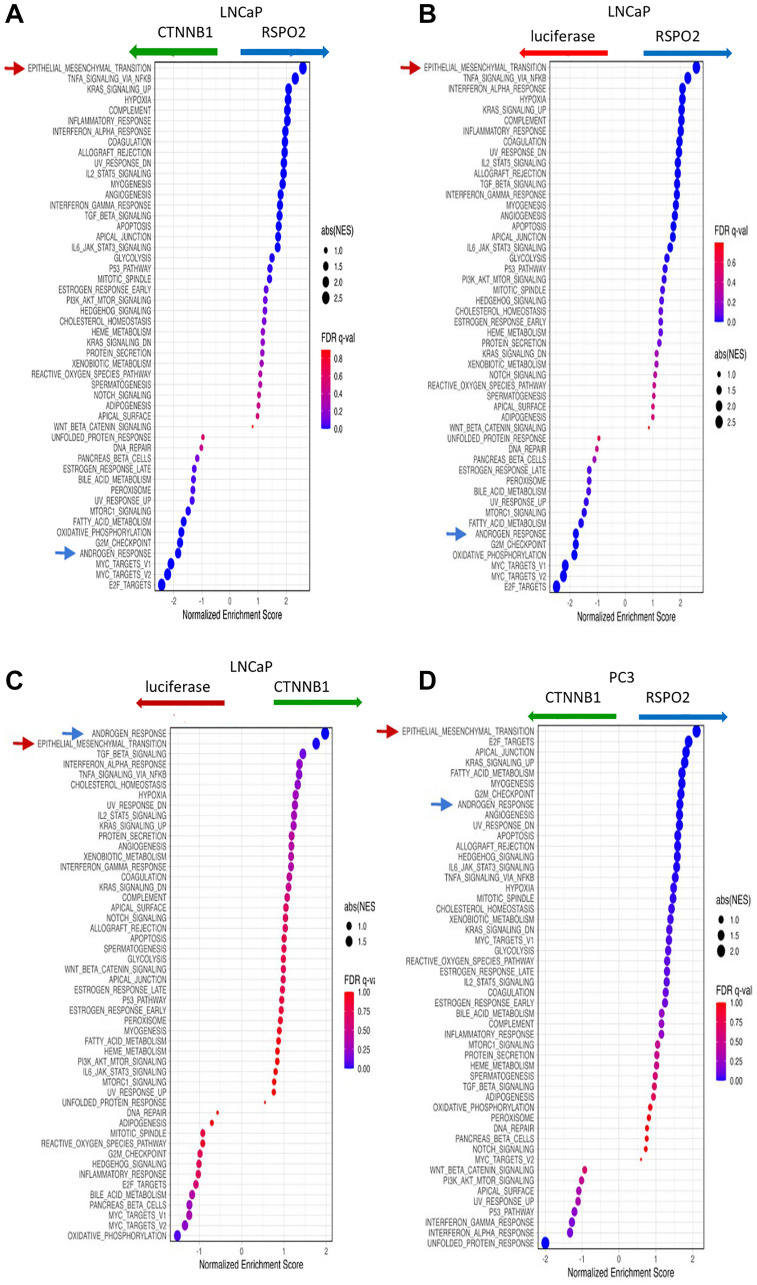
50 Hallmark pathways were analyzed by GSEA, in which we show the differences when directly comparing LNCaP cells that express (**A**) RSPO2 or CTNNB1, (**B**) RSPO2 or LUC, (**C**) CTNNB1 to LUC. (**D**) The same signatures were analyzed by GSEA in PC3 cells that express RSPO2 to CTNNB1. In all GSEA, the EMT pathway (red arrow) and Androgen Response pathways (blue arrow) are highlighted.

Using gene set enrichment analysis (GSEA) [21], we demonstrated that the EMT signature was consistently upregulated in *RSPO2* overexpressed cells in both AR+ and AR- cells, while androgen response was consistently downregulated in AR+ cells, but not in AR- cells (Figure 6A–6D).

### RSPO2 is positively correlated with genes that define AR-subtypes of PC

**Figure 7:**
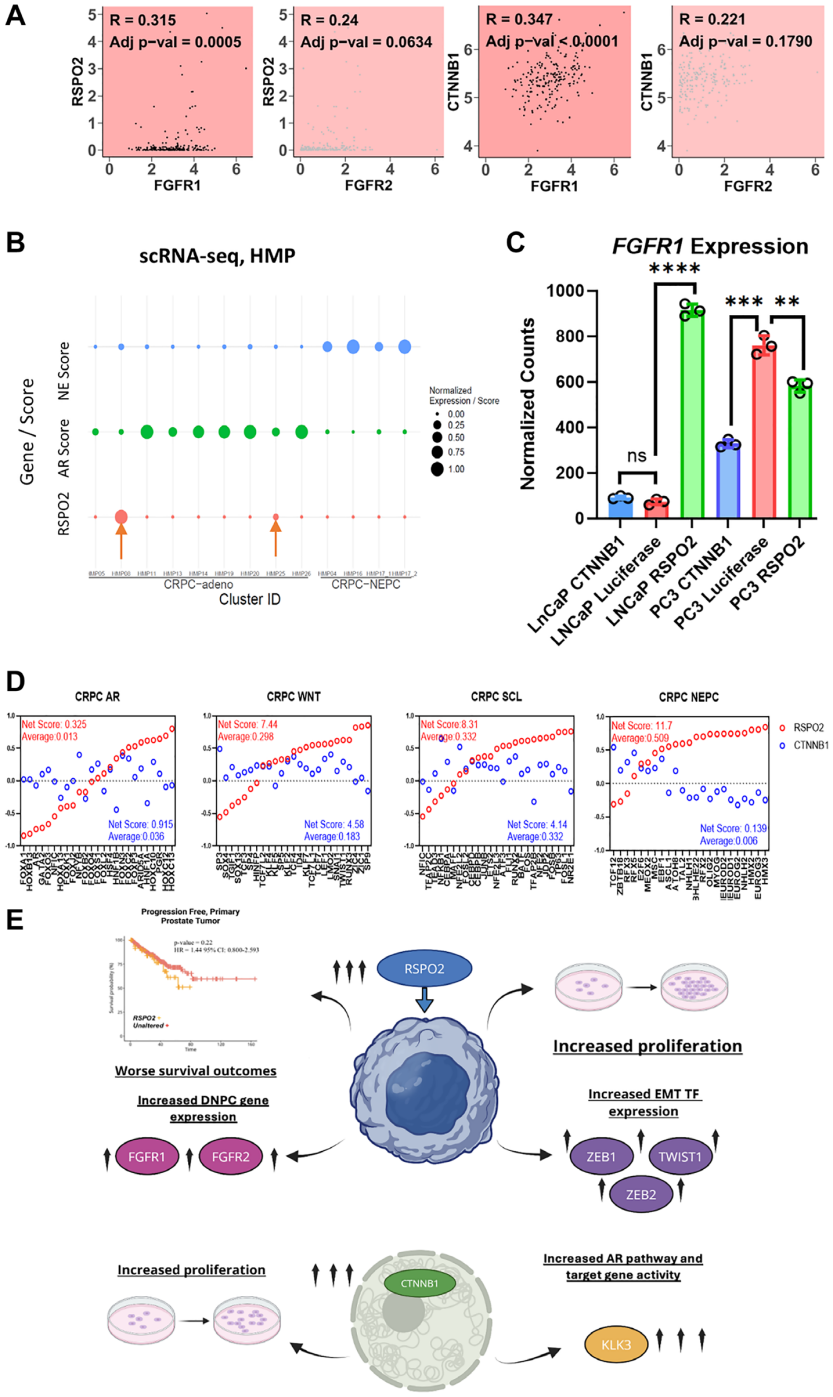
(**A**) In 208 SU2C CRPC samples, Pearson correlations compare the transcript expression of *RSPO2* or *CTNNB1* compared to *FGFR1/2*. Pearson correlation coefficient and adj *p*-values are shown. (**B**) A bubble plot is used to depict single cell RNA-seq data acquired from 12 prostate cancer samples to evaluate *RSPO2* expression with respect to relative AR or NEPC activity. AR positive and negative samples are annotated as CRPC-adeno and CRPC-NEPC respectively. (**C**) Normalized counts for FGFR1 expression are shown. (**D**) Of the transcriptional profiles from Tang et al. [23] we examined ALAN associations of RSPO2 (red) or CTNNB1 (blue) with the 4 categories of transcription factors that were classified as CRPC-AR, CRPC-WNT, CRPC-SCL, or CRPC-NEPC. The ALAN interaction scores range between -1 to 1, in which we depict the sum and average interaction for each of the 25 transcription factors. (**E**) A diagram summarizing the impact of *RSPO2* overexpression is shown.

To further explore the role of RSPO2 in mCRPC, we focused on the analysis of its association with FGFRs, which are known drivers of DNPC [18]. Through co-expression patterns in mCRPC samples from SU2C, *RSPO2* strongly correlated with *FGFR1/2* (R = 0.315 adj. *p*-value = 0.0005, R = 0.24 adj. *p*-value = 0.0634, respectively) (Figure 7A). Next, we evaluated single-cell RNA-seq data from CRPC patients to determine the relationship of *RSPO2* expression with respect to the AR or neuroendocrine activity scores of the 12 samples. In doing so, we observed two patients with increased *RSPO2* expression, both of whom had coordinate reduction of AR and neuroendocrine prostate cancer (NEPC) activity based on prior gene signatures [22] (Figure 7B). In our AR+ cell line, *RSPO2* overexpressed cells also yielded an increased expression of *FGFR1* compared to the *CTNNB1* overexpressing or negative control cells (Figure 7C). In a recent study, Tang et al. subtyped mCRPCs into four major classes based on the relative activity of 25 transcription factors in each group [23], which yielded tumors that were AR-driven, Wnt-active, Stem cell-like (SCL), or NEPC-like. In the 208 mCRPC tumors from SU2C, we computed ALAN interactions with either *RSPO2* or *CTNNB1* with respect to each of the 25 transcription factors belonging to the four subclasses of mCRPC. It was clear that *CTNNB1*, the known Wnt transcription factor, had overall similar behavior as Wnt or SCL transcription factors, but not the AR or NEPC transcription factors. *RSPO2* is distinctly associated with all but the AR transcription factors, and exhibited generally great similarities to these transcriptional programs as compared to *CTNNB1* (Figure 7D). A schematic was generated using RSPO2 and CTNNB1, summarizing both the transcriptional and proliferative differences between RSPO2 and CTNNB1 (Figure 7E). Altogether, *RSPO2* expression was associated with FGFRs, and overexpression in AR+ cell lines led to increases in *FGFR1*/*2*. Further, supported by our modeling approaches in mCRPCs, RSPO2 exhibited strong associations with numerous transcription factors in AR-mCRPCs.

### Supplemental

Cells that are supposed to be overexpressing *RSPO2* and *CTNNB1* display significant overexpression as compared to the other cell lines (Supplementary Figure 1A, 1B). Overexpression of RSPO2 led to decreased expression of epithelial marker *CDH1*, but increased the expression levels of mesenchymal markers *CDH2* and *VIM* (Supplementary Figure 1C–1E). RSPO2 overexpressed cells showed decreased expression of the known AR target *KLK3* (Supplementary Figure 1F).

## DISCUSSION

In this study, we conducted a comprehensive analysis of *RSPO2* in PC using a combination of bioinformatic approaches and functional assays in cell lines. We analyzed data from both primary PC and mCRPC patients to investigate the prevalence of *RSPO2* alterations compared to other RSPO family members and Wnt oncogenes. Our findings revealed that *RSPO2* alterations occurred more frequently than those in other family members, as well as canonical Wnt regulators *CTNNB1* and *APC*, providing insight into its potential significance in prostate cancer progression. Additionally, we examined the effects of *RSPO2* overexpression in both androgen receptor-positive (AR+) and androgen receptor-negative (AR-) prostate cancer cell lines. While we observed significant increases in cell proliferation, RSPO2 was more proficient than CTNNB1 in increasing the expression of genes in the Hallmark EMT pathway and two critical transcription factors, *ZEB1* and *TWIST1*. We also investigated and uncovered RSPO2’s lack of correlation with AR through RNA analysis and computational modeling. Furthermore, our computational modeling approaches also indicated the distinction of RSPO2 with other RSPO family members, but also indicated that RSPO2 was strongly associated with several mCRPC transcription factors thought to drive AR-mCRPCs. Pending future investigations, RSPO2 appeared to both associate with and regulate traits found in AR-DNPCs. Altogether, increases in RSPO2 in PC warrant significant attention in both the clinical and laboratory settings, as this secreted molecule appears to sufficiently promote various features that would drive resistance to standard-of-care systemic therapies.

Despite the implications of RSPO2 as a pro-tumor Wnt signaling regulator, there is no current effort to directly disrupt RSPO2 signaling in the clinical setting. To our knowledge, there are no FDA-approved drugs that are added routinely into clinical trials that target the Wnt pathway [24], nor are there any RSPO2 inhibitors that have been granted FDA approval for cancer treatments. Credentialing RSPO2 as a pharmacological target in part requires knowledge that disambiguates RSPO2 from other family members. The family of RSPO proteins is thought to be functionally redundant proteins that promote Wnt pathway signaling [25], an observation supported by our ALAN gene behavior analysis. However, *RSPO2* amplification rates supersede each of the other family members in PC and across cancer types. Further, based on the predicted protein structures of RSPO2 and family members, nuanced differences may allow for the development of a pharmacological reagent with selectivity against RSPO2. One therapeutic modality of RSPO2 inhibition could be through antibodies, which would theoretically be effective against secreted or extracellular factors [26]. In particular, a blocking antibody has shown efficacy against acute myeloid leukemia cells [27]. However, in that study, the authors indicate that RSPO2 blockade mainly attenuated autocrine BMP signaling, which indicates that the function of RSPO2 may extend beyond regulation of Wnt. Regardless, our findings support the need for the development of therapeutics targeting RSPO2, which is projected to attenuate signaling that drives the progression of PC.

Based on our transcriptional profiling performed on cell lines with *RSPO2* overexpression, RSPO2 appeared to have an immediate effect on DNPC and EMT genes. While RSPO2 has been implicated in EMT [16], it has not been directly linked to the expression of transcription factors of EMT, including *ZEB1* and *TWIST1* [17]. These results were supported by the observations in mCRPC samples, in which we found that *RSPO2* exhibited similar ALAN behavior profiles as a suite of EMT transcription factors. Surprisingly, this effect was not observed through *CTNNB1* overexpression in the same setting. Based on our GSEA analysis, Hallmark EMT was consistently the most pronounced pathway upon *RSPO2* overexpression, even when directly compared to *CTNNB1* overexpression and in the AR-PC3 cell line. Here, *CTNNB1* overexpression also promoted proliferation, but instead yielded increases in Hallmark androgen activity as the top pathway, with EMT ranking second. We note that RSPO2 led to significant decreases in Hallmark androgen activity, which supports the findings that, in mCRPCs, RSPO2 was associated with three of the four mCRPC subtypes thought to be driven by non-AR activity [23]. In that seminal study, they indicated that the stem cell-like (SCL) CRPCs were the second most common subtype of CRPCs. Finally, we note that while RSPO2 also promoted proliferation and Hallmark EMT in AR-PC3 cells, it did not increase the expression of *ZEB1* or *TWIST1*, perhaps due to the elevated baseline expression of these genes. While much more is required to define RSPO2 as a driver of mCRPC, our findings certainly justify a need to consider this secreted factor as a potential driver of aggressive AR-subtypes. On a grander scheme, it is also intriguing that a secreted factor has such a pronounced impact on Hallmark EMT, which is consistently enriched in metastatic PC [19, 20].

Bluemn et al. [18] pioneered the concept of DNPCs as a treatment-emergent subtype of mCRPC. DNPCs are thought to lack androgen receptor activity and do not present with NEPC markers. These tumors are largely thought to be driven by the signaling of FGFRs, in which they demonstrated that DNPC cell models and tumors exhibited elevated levels of the FGFRs. Besides inhibiting androgen receptor activity, this seminal work never indicated other approaches that may yield such tumors. Our ALAN gene behavior analysis indicates that *RSPO2* was correlated with *FGFR1* and *FGFR2*, while exhibiting anti-correlation with *AR* or its co-regulators. Interestingly, our cell models also supported that RSPO2 increased *FGFR1* expression, but only in the AR+ LNCaP cell lines. Again, this was not observed by *CTNNB1* overexpression. While it is tempting to nominate RSPO2 as a driver of DNPCs, there are still nuances that may not support this thesis. As one example, our ALAN analysis in mCRPCs also indicated an association of *RSPO2* with NEPC transcription factors, including ASCL1 [28]. Altogether, there is a need to functionally explore RSPO2 in differentiation models of prostate cancer, which, to our knowledge, may be sparse.

In conclusion, our study establishes RSPO2 as a clinically relevant prognostic marker with pro-tumor functions. We have indicated that RSPO2 may have different properties from other RSPO family members and Wnt regulators such as CTNNB1, and therefore needs to be examined independently. As a secreted factor, interrogation of RSPO2-blocking antibodies or analogous reagents could be a promising avenue to treat mCRPCs that are resistant to therapies that block AR signaling. Further, future mechanistic studies may indicate that RSPO2 has additional functions outside of promoting Wnt signaling.

## MATERIALS AND METHODS

### Cell generation and maintenance

Two human PC lines, LNCaP and PC3, were cultured in RPMI 1640 media (Gibco). Media were supplemented with 10% FBS (R&D Systems), 1% Penicillin (Gibco) and Streptomycin (Gibco), and 0.2% Glutamax (Gibco). Both cell lines were ordered from the American Type Culture Collection (ATCC). These cells were passaged and tested for fewer than 6 months after ordering and were authenticated by ATCC using STR profiling. To produce cell lines overexpressing *CTNNB1*, luciferase, and *RSPO2*, a mammalian lentiviral expression vector (Vectorbuilder, VB241114-1397jxm, ID:VB240917-1425 tbq, ID:VB240729-1427prc) was used to infect each cell line. This was done in the presence of 10 μg/ml polybrene (Gibco) and then selected by multiple rounds of 10 μg/ml puromycin (Gibco).

### Cell proliferation experiments

In six-well plates, 15,000 cells were plated with 2 mL of media. The cells were harvested and counted on days 5, 7, and 10 of experimentation. The cells were counted using a Corning Cell Counter and CytoSMART software. In addition to counting on day 5, 0.5 mL of media were added to each well.

### RNA-sequencing experiments

RNA was isolated for sequencing using the RNeasy Mini Kit (Qiagen) following manufacturer recommendations. For the RNA-seq experimentation, library preparation, quality control, and sequencing, prepared samples were shipped to Novogene.

### RNA expression analysis

The raw read counts were imported into R (version 4.3.3). The raw reads were then filtered to only include genes that were expressed in at least one sample. The data were converted to VST normalized counts using DESeq2 (1.42.1), which was used to control for cross-sample normalization based on library size. The adaptive shrinkage estimator from the “ashr” package [29] was used to add shrunken log2 fold changes and their standard errors. EnhancedVolcano (1.13.2) was used on these objects to create the volcano plots. To create the boxplots, the normalized count matrix was used. To determine statistical significance, a Wilcoxon rank-sum test was performed between samples, with corrections for multiple comparisons done using the Benjamini-Hochberg method to control the FDR at a significance level of 0.05. Raw reads were trimmed, aligned to the GRCh38 human genome, and gene-level read counts were generated using the CHURP [30] pipeline.

### Gene set enrichment analysis (GSEA)

We conducted GSEA (4.3.3) based on 50 hallmark gene sets from the MSigDB database [31]. From this, normalized enrichment scores (NES) were obtained and used to compare pathway enrichment across the different conditions.

### Structure prediction of RSPO-family proteins using AlphaFold

FASTA protein sequences for the RSPO-family were obtained from NCBI with the following sequence IDs: NP_001229837.1 (RSPO1), NP_848660.3 (RSPO2), NP_116173.2 (RSPO3), NP_001025042.2 (RSPO4). The RSPO-family protein sequences were aligned using the EMBL-EBI ClustalW multiple sequence alignment program. Structural predictions for each RSPO-family protein were generated using AlphaFold2 (version 2.3.1-multimer). The model with the highest average predicted local distance difference test (pLDDT) score, which estimates per-residue modeling confidence, for each individual RSPO-family protein was used for visualization using PyMOL (2.5.0). The PyMOL ‘super’ command was used to superimpose RSPO2 with the other RSPO-family proteins and calculate the average RMSD between models. Comparisons were made for the entire protein model. A ray-traced image (ray_trace_mode, 1; ray_trace_color, black; ray_opaque_background, off; ray_shadows, 0) of each RSPO-family protein was created for visualization. These data processing steps were conducted using the Minnesota Supercomputing Institute.

### ALAN analyses

The Algorithm for Linking Activity Networks (ALAN) was performed on the SU2C dataset. Relevant genes were pulled from this output and visualized in a heatmap, which employed unsupervised hierarchical clustering to group the genes. ALAN profiles for each of the genes were extracted and shown in the form of violin plots. Uniform Manifold Approximation and Projection (UMAP) was applied on the SU2C dataset ALAN outputs using the umap R package (version 0.2.10.0) with the default parameters.

### Pearson correlation scatter plots

Pearson correlations were calculated and scatter plots were generated in R using ggplot2 (version 3.5.1) to visualize the relationship between a given pair of genes. Linear regression lines were fitted to the data to highlight the strength and direction of these relationships. *P*-values were adjusted for multiple comparisons using the Benjamini-Hochberg method to control the false discovery rate (FDR) at a significance level of α = 0.05.

### Multiple sequence alignment/hydropathy plot

FASTA protein sequences were obtained for the RSPOS from NCBI. Using the Basic Local Alignment Search Tool (BLAST), sequencing was conducted on RSPO2 (NP_848660.3) with reference to RSPO1 (NP_001229837.1), RSPO3 (NP_116173.2) and RSPO4 (NP_001025042.2). The alignment was scored and colored by TCoffee’s TCS program, with the color indicating the local reliability. The hydropathy plot was generated using the BLAST sequence viewer and then coloring based on side chain hydropathy which has been correct for solvation [32].

## SUPPLEMENTARY MATERIALS



## Abbreviations

PC: Prostate Cancer
AR: Androgen Receptor
DNPC: Double Negative Prostate Cancer
mCRPCs: metastatic prostate cancers
NEPC: Neuroendocrine prostate cancer
EMT: Epithelial-Mesenchymal Transition
ADT: Androgen Deprivation Therapy
TCGA: The Cancer Genome Atlas
SU2C: Stand Up 2 Cancer
SCL: Stem Cell Like
RMSD: Root Mean Square Deviation
PSA: Prostate Specific Antigen
