# Supplementary material for: Dissecting the functional differences and clinical features of R-spondin family members in metastatic prostate cancer
Source: Oncotarget. 2025 Jul 25;16:606–20. doi: 10.18632/oncotarget.28758 (PMC12406459; doi:10.18632/oncotarget.28758)
Supplement: Supplementary file 1 [file oncotarget-16-28758-s001.pdf]

Dissecting the functional differences and clinical features of R-spondin family members in metastatic prostate cancer

SUPPLEMENTARY MATERIALS

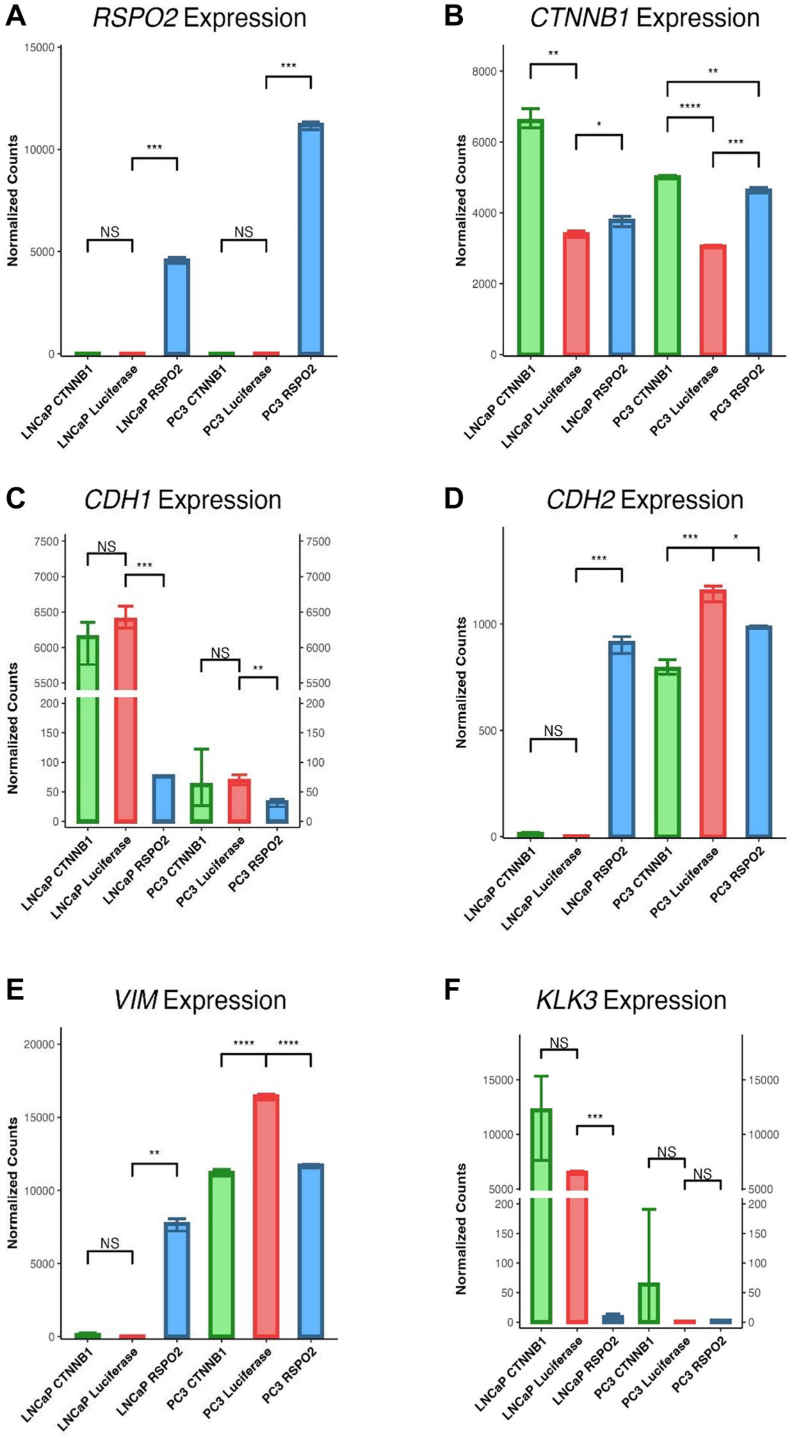

Supplementary Figure 1: The normalized counts for the relative expression of *RSPO2* overexpressed AR+ and AR- cell lines is shown for (A) *RSPO2* **(B)** *CTNNB1* **(C)** *CDH1* **(D)** *CDH2* **(E)** *VIM* **(F)** and *KLK3*. Statistical comparisons were conducted using Welch's two sample t-tests, with $^{*}p < 0.05$, $^{**}p < 0.01$, $^{***}p < 0.001$, $^{****}p < 0.0001$.
